# Supplementary material for: The effects of introgression across thousands of quantitative traits revealed by gene expression in wild tomatoes
Source: PLoS Genet. 2021 Nov 8;17(11):e1009892. doi: 10.1371/journal.pgen.1009892 (PMC8601620; doi:10.1371/journal.pgen.1009892)
Supplement: S1 Table — (DOCX) [file pgen.1009892.s004.docx]

|  | **Pattern of expression similarity** | | | |  |
| --- | --- | --- | --- | --- | --- |
| **Gene tree topology** |  | P1P2 | P1P3 | P2P3 | Sum |
|  | P1P2 | 4249 (4245.1) | 3450 (3450.5) | 3193 (3196.4) | 10892 |
|  | P1P3 | 43 (42.9) | 32 (34.8) | 35 (32.28) | 110 |
|  | P2P3 | 19 (23.0) | 22 (18.7) | 18 (17.3) | 59 |
|  | Sum | 4311 | 3504 | 3246 |  |
