# Supplementary material for: The effects of introgression across thousands of quantitative traits revealed by gene expression in wild tomatoes
Source: PLoS Genet. 2021 Nov 8;17(11):e1009892. doi: 10.1371/journal.pgen.1009892 (PMC8601620; doi:10.1371/journal.pgen.1009892)
Supplement: S2 Table — (DOCX) [file pgen.1009892.s005.docx]

|  | **Pattern of expression similarity** | | | |  |
| --- | --- | --- | --- | --- | --- |
| **Gene tree topology** |  | P1P2 | P1P3 | P2P3 | Sum |
|  | P1P2 | 3237 (3236.3) | 2821 (2809.3) | 3149 (3161.4) | 9207 |
|  | P1P3 | 223 (191.9) | 150 (166.59) | 173 (187.5) | 546 |
|  | P2P3 | 428 (459.8) | 404 (399.1) | 476 (449.1) | 1308 |
|  | Sum | 3888 | 3375 | 3798 |  |
