# Supplementary material for: The effects of introgression across thousands of quantitative traits revealed by gene expression in wild tomatoes
Source: PLoS Genet. 2021 Nov 8;17(11):e1009892. doi: 10.1371/journal.pgen.1009892 (PMC8601620; doi:10.1371/journal.pgen.1009892)
Supplement: S1 Text — (DOCX) [file pgen.1009892.s013.docx]

**Supplementary Materials and Methods for Hibbins & Hahn 2021**

**Derivation of expected trait variances and covariances under the multispecies network coalescent**

­As discussed in the methods, we assume a rooted three-taxon tree with the topology ((A,B),C) (Figure 1). In coalescent units of *2N* generations, A and B speciate at time *t*_1_, and C speciates from the ancestor of A and B at time *t*_2_. An instantaneous introgression event between species B and C occurs at time *t*_m_. This may occur from C into B at a rate of δ_2_, and/or from B into C at a rate of δ_3_. These rates denote the probability that a locus has the specified introgression history. Each possible history generates a “parent tree” for the locus, within which incomplete lineage sorting occurs according to the multispecies coalescent model. We denote these parent trees 1, 2, and 3 for the history of no introgression, C into B introgression, and B into C introgression, respectively.

For each pair of species, the expected phylogenetic covariance is the weighted average of expected internal branch lengths across all gene trees where that species pair shares a subtending internal branch. This gives the following general expression:

$$E\left[ cov\left( X,Y \right) \right]=\sum_{\tau_{s}} p_{h}p\left( \tau_{s,i} \right)L_{internal}\left( \tau_{s,i} \right) [1]$$

Where *p_h_* is the probability of the parent tree relevant to the gene tree of interest, $p\left( \tau_{s,i} \right)$ is the probability of the gene tree $\tau_{s,i}$, and $L_{internal}\left( \tau_{s,i} \right)$ is the expected internal branch length of that gene tree. This product is summed across the subset of gene trees $\tau_{s}$ where species X and Y share an internal branch.

For three taxa, the exact expectations for these gene tree frequencies, and expected coalescence times within them, are known (Hibbins and Hahn 2019). The expected internal branch lengths are then simply the difference of the two expected coalescence times for each gene tree. We begin with the species pair AB, which share an internal branch in four gene trees: one the result of lineage sorting at non-introgressed loci, and the others the result of incomplete lineage sorting in each of the three possible parent trees. These gene trees will be denoted *AB*1_1_, *AB*2_1_, *AB*_2_, and *AB*_3_. In this notation, the subscript denotes the parent tree from which the gene tree originates, while the non-subscripted number indicates whether the tree is the result of lineage sorting (1) or incomplete lineage sorting (2) for topologies where both are possible. For the first two terms, we have:

$$p\left( AB1_{1} \right)=1-e^{-\left( t_{2}-t_{1} \right)} [2]$$

$$L_{internal}\left( AB1_{1} \right)=\left[ t_{2}+1 \right]-\left[ t_{1}+\left( 1-\frac{t_{2}-t_{1}}{e^{t_{2}-t_{1}}-1} \right) \right]= \frac{e^{t_{2}}\left( t_{2}-t_{1} \right)}{e^{t_{2}}-e^{t_{1}}} [3]$$

$$p\left( AB2_{1} \right)=\frac{1}{3}e^{-\left( t_{2}-t_{1} \right)} [4]$$

$$L_{internal}\left( AB2_{1} \right)=\left[ t_{2}+\frac{1}{3}+1 \right]-\left[ t_{2}+\frac{1}{3} \right]=1 [5]$$

Note in equation 5 that the expected internal branch length is 1 coalescent unit. This holds true for any gene tree produced by incomplete lineage sorting. For gene tree *AB*_2_, which comes from a history of C into B introgression, the lower bound to coalescence is *t*_m_ rather than *t*_1_, giving the following:

$$p\left( AB_{2} \right)=\frac{1}{3}e^{-\left( t_{2}-t_{m} \right)} [7]$$

$L_{internal}\left( AB_{2} \right)=1 [8]$

In parent tree 3 (B into C introgression), C traces its ancestry through B going backwards in time. This allows C to coalesce with A after *t*_1_, rather than *t*_2_. For the gene tree *AB*_3_, this gives:

$$p\left( AB_{3} \right)=\frac{1}{3}e^{-\left( t_{1}-t_{m} \right)} [9]$$

$$L_{internal}\left( AB_{3} \right)=1 [10]$$

By applying equations 2-10 to the general formulation of equation 1 (and then simplifying), we obtain the expected trait covariance between A and B (equation 6 of the main text).

For the species pair BC, there are five gene trees where the internal branch is shared. Two are the result of lineage sorting at introgressed loci, and the others are the result of incomplete lineage sorting at either introgressed or non-introgressed loci. These will be denoted *BC*1_2_, *BC*1_3_, *BC*2_2_, *BC*2_3_, and *BC*_1_, respectively. The expected gene tree frequencies and branch lengths often mirror those for the AB species pair, but with *t*_m_ in the place of *t*_1_. From this we obtain the following equations:

$$p\left( BC1_{2} \right)=1-e^{-\left( t_{2}-t_{m} \right)} [11]$$

$$L_{internal}\left( BC1_{2} \right)=\left[ t_{2}+1 \right]-\left[ t_{m}+\left( 1-\frac{t_{2}-t_{m}}{e^{t_{2}-t_{m}}-1} \right) \right]=\frac{e^{t_{2}}\left( t_{2}-t_{m} \right)}{e^{t_{2}}-e^{t_{m}}} [12]$$

$$p\left( BC1_{3} \right)=1-e^{-\left( t_{1}-t_{m} \right)} [13]$$

$$L_{internal}\left( BC1_{3} \right)=\left[ t_{1}+1 \right]-\left[ t_{m}+\left( 1-\frac{t_{1}-t_{m}}{e^{t_{1}-t_{m}}-1} \right) \right]=\frac{e^{t_{1}}\left( t_{1}-t_{m} \right)}{e^{t_{1}}-e^{t_{m}}} [14]$$

$$p\left( BC2_{2} \right)=\frac{1}{3}e^{-\left( t_{2}-t_{m} \right)} [15]$$

$$p\left( BC2_{3} \right)=\frac{1}{3}e^{-\left( t_{1}-t_{m} \right)} [16]$$

$$p\left( BC_{1} \right)=\frac{1}{3}e^{-\left( t_{2}-t_{1} \right)} [17]$$

$$L_{internal}\left( BC2_{2} \right)=L_{internal}\left( BC2_{3} \right)=L_{internal}\left( BC_{1} \right)=1 [18]$$

Applying equations 11 through 18 to equation 1 and then simplifying, we obtain the expected trait covariance between B and C (equation 7 of the main text).

Finally, for the species pair AC, there are three possible gene trees where these species share an internal branch, produced by incomplete lineage sorting within each of the three parent trees (since we do not model introgression between this pair here). These are dubbed *AC*_1_, *AC*_2_, and *AC*_3_ for the trees produced by parent trees 1, 2, and 3, respectively. For the expected gene tree frequencies and branch lengths, we have:

$$p\left( AC_{1} \right)=\frac{1}{3}e^{-\left( t_{2}-t_{1} \right)} [19]$$

$$p\left( AC_{2} \right)=\frac{1}{3}e^{-\left( t_{2}-t_{m} \right)} [20]$$

$$p\left( AC_{3} \right)=\frac{1}{3}e^{-\left( t_{1}-t_{m} \right)} [21]$$

$$L_{internal}\left( AC_{1} \right)=L_{internal}\left( AC_{2} \right)=L_{internal}\left( AC_{3} \right)=1 [22]$$

Equations 19-22 applied to equation 1 and then simplified give us the expected trait covariance between A and C (equation 8 of the main text).

Finally, we consider the expected trait variance. Under Brownian motion, this variance is simply the total height of the tree from the root to the relevant tip. To obtain the expected variance with introgression, we take the sum of this height across all gene trees, weighted by their probability. Concordant trees produced by lineage sorting have a total height of *t*_2_ + 1, and all other gene trees have a height of *t*_2_ + 1 + 1/3. Owing to this consistency in expected gene tree heights, all three species have the same expected trait variances. To get these heights we have to consider the contribution of gene trees from all three parent trees. For parent tree 1, this variance is:

$$E\left[ var\left( Gene trees|parent tree 1 \right) \right]=\left[ \left( 1-e^{-\left( t_{2}-t_{1} \right)} \right)\left( t_{2}+1 \right) \right]+\left[ \left( e^{t_{2}-t_{1}} \right)\left( t_{2}+1+1/3 \right) \right] [23]$$

For parent tree 2:

$$E\left[ var\left( Gene trees|parent tree 2 \right) \right]=\left[ \left( 1-e^{-\left( t_{2}-t_{m} \right)} \right)\left( t_{2}+1 \right) \right]+\left[ \left( e^{t_{2}-t_{m}} \right)\left( t_{2}+1+1/3 \right) \right] [24]$$

And for parent tree 3:

$$E\left[ var\left( Gene trees|parent tree 3 \right) \right]=\left[ \left( 1-e^{-\left( t_{1}-t_{m} \right)} \right)\left( t_{1}+1 \right) \right]+\left[ \left( e^{t_{1}-t_{m}} \right)\left( t_{1}+1+1/3 \right) \right] [25]$$

Summing equations 23-25, weighted by the probabilities of their respective parent trees, gives equation 9 of the main text, the expected trait variance of the three species.
