# Supplementary material for: The effects of introgression across thousands of quantitative traits revealed by gene expression in wild tomatoes
Source: PLoS Genet. 2021 Nov 8;17(11):e1009892. doi: 10.1371/journal.pgen.1009892 (PMC8601620; doi:10.1371/journal.pgen.1009892)
Supplement: Attachment — Submitted filename: intro_expression_responses.docx [file pgen.1009892.s014.docx]

This manuscript has been reviewed thoroughly by three referees, all of whom find the work interesting and compelling. The reviews offer suggestions for improvement of the manuscript, including some requests for possible additions to the analyses, which I encourage the authors to consider. Reporting a false positive rate along with the power analysis seems like a good idea. The reviews are also very clear in their praise for the clarity of the presentation and the important contribution this work will make to how we think about and analyze trait covariation across species. I agree and think this manuscript will be read with lots of interest.

We are grateful to the editor and reviewers for their optimistic and constructive comments. We have added new sets of conditions to the power analysis and made changes to the text to address all comments. We have detailed our changes below in red.

As a minor point, I will add that it would be good to emphasize the time scale a bit more in the introduction. For example, on line 92, perhaps "To address the effects of introgression on quantitative traits" could be modified to "To address the effects of historical introgression on quantitative traits". I think this is worthwhile because people study trait variation due to contemporary admixture and introgression regularly with admixture mapping. The text is clear that this manuscript is about phylogenomics, deeper timescales reflected on trees, and comparative methods, but I believe readers will be benefit from a very explicit statement in the introduction of the relevant time scale.

We now emphasize the historical scale of introgression in the Introduction both when we define introgression (line 52) and on line 92:

“*Introgression—the historical hybridization and subsequent backcrossing of previously isolated lineages …*”

“*To address the effects of historical introgression on quantitative traits …*”

Reviewer's Responses to Questions

**Comments to the Authors:
Please note here if the review is uploaded as an attachment.**

Reviewer #1: The paper by Hibbins and Hahn proposes a statistical framework for estimating the amount of introgression between two species while accounting for incomplete lineage sorting (ILS). Both ILS and introgression are common in evolution and failure to account for them can influence inferences of the rate, degree of convergence, or amount of selection that has occurred on a particular trait. As such, this work is an important step towards a more complete understanding of the evolution of quantitative traits. The paper is clearly written and the results presented in an easy to follow manner. However, I do have a couple of concerns about the current presentation.

1. The authors did not present data on false positive rates and this information is crucial for understanding the power analyses performed. This data should already be included in the simulations, so additional simulations aren’t needed.

2. The power analysis comparing a model of ILS only and of ILS + introgression used a single set of parameters and the values chosen for some parameters (e.g. number of genes and introgression amount) are more conducive to identifying a signal of introgression than what is presented in the power analysis of just the ILS+introgression model or the empirical data analyzed. It would be useful to see the results of this comparison for a range of conditions, especially those that covered what would be expected for the empirical data to better understand the ability to distinguish between these two models.

In response to these two comments, we have added new simulations to the power analysis under a no-introgression condition, varying the rate of ILS and the number of traits. We found a low false positive rate (< 5%) in all conditions. These results are presented in Supplementary Figure 2, and discussed in the text on lines 280-281.

3. It is hard to tell exactly, but the power analyses performed suggest relatively low power to detect introgression in the empirically analyzed data using the ILS+introgression approach. This is particularly true for the ‘low’ data set, yet introgression is found. A discussion or resolution of this discrepancy would be useful to readers.

We have added a new paragraph to the Discussion that addresses this issue (lines 417-426):

“*Our power analysis suggests that we should have had low power to detect an effect of introgression in the low triplet, which has a rate of introgression of less than 1%. One explanation for the fact that we do detect an effect is that the introgressed variation in this triplet affects the downstream regulation of a large set of correlated genes, though the discussion in the previous paragraph likely rules out this possibility. Very recent introgression is also an unlikely explanation, as our power analysis shows that the timing of introgression does not have an effect at low rates. As previously discussed, directional selection on introgressed variation in the low triplet could also improve the power; evaluating this possibility would be an interesting future direction. Finally, we may simply have been fortunate to observe a positive result, even with reduced (but non-zero) power in this area of parameter space.*”

4. The presented Q3 statistic allows one to estimate the extent and species involved in introgression. However, as currently presented it is not integrated into a framework that allows the estimation of the rate of trait evolution or the degree of ILS. Maybe I’m mistaken, but the models seem to be nested, with the ILS only model being a special case of the ILS+introgression model. This would suggest that a likelihood ratio approach could be used to determine whether the inclusion of introgression in the model is actually warranted by the data. I recognize that this was not the approach taken by the authors, but this seems like a limitation to the current approach that would be useful to mention.

In some sense, we are evaluating whether an ILS-only or ILS+introgression model is a better fit to the data when we test whether Q3 is significantly different from 0. We do agree that, as a summary statistic, Q3 is limited in the degree of information it can provide, as it does not directly estimate the rate of evolution or degree of ILS (there are other statistics better suited for these tasks). Our hope is that our work helps spark the development of comparative methods that can perform more sophisticated analyses, such as the sort of likelihood ratio test the reviewer describes. We now emphasize this in the last paragraph of the Discussion (lines 469-471):

“… *Future phylogenetic comparative approaches should strive to evaluate the contributions of both ILS and/or introgression to trait evolution, allowing for more accurate evolutionary inferences.*”

Minor point
Line 482 – missing location of script

We have now added the missing link (now on lines 500-501).

Reviewer #2: This paper develops an approach to examine gene expression variation and covariation between species that takes into account both incomplete lineage sorting and introgression. The paper then goes to show that gene expression covariation between species that have been subject to introgression also show the effect in gene expression. They show this in particular within 2 subclades in Solanum that show varying levels of introgression in the genome.

The modelling seems sound although I do not have the expertise to comment on this deeply. However, I do think this is a very interesting area of study as we begin to grasp the multilayered effects of interspecies hybridization on evolution. The results with gene expression data do show a significant effect of introgression, although the magnitude of the difference was not great (476 vs. 449 genes).

My one concern is that the authors really only apply their method to 2 groups – the low and high triplet clades they identify. It would have been much stronger if they could find other comparisons, as there will always be a question of whether this particular high triplet is a fluke. Conversely, maybe showing what the results look like in clades that have no evidence of introgression may also provide greater confidence.

We agree that our results would be bolstered by testing more triplets; unfortunately, the data for carrying out such tests is currently quite limited. We had carried out a search when starting this project and found few suitable datasets. A search carried out again in the past month turned up no additional datasets, especially where both gene expression data and gene tree discordance data is available and easy to integrate. We now address this in a new paragraph in the Discussion (lines 426-433):

“… *Distinguishing random chance from other processes would be facilitated by testing additional triplets; unfortunately, we have exhausted the independent triplets possible from our data, having used six of the eight available accessions with gene expression data. Very few multispecies transcriptomic datasets are currently available in systems with widespread introgression, though similar tests may be possible from data in the butterfly genus, Heliconius (Catalán et al. 2018, Catalán et al. 2019). Analyzing or generating such datasets in other systems would help to confirm the generality of our findings.*”

Reviewer #3: The evolution of gene expression has been an important source of research in the last three decades. Nonetheless, most, if not all studies of gene expression, ignore the effect that introgression might have on the distribution of gene expression as a trait in phylogenetic trees. The first part of the paper addresses this very gap and develops an analytical framework to recapitulate the evolution of quantitative traits following a Brownian motion model of evolution in the face of introgression and incomplete lineage sorting. The piece provides a derivation of the expected trait variances and covariances among hybridizing species using the multispecies network coalescent as a framework. The methods are clear, the appendix is welcome and the results are well presented. The authors are forward and state that while are other models of trait evolution but elaborate on the reasons to not expand their model. The model is elegant and timely and I think will be an excellent addition to the literature. The authors present a fairly large simulation analyses in Figure 3 which also bolster the importance of the piece.
The second part of the paper takes the theoretical prediction and uses the model to evaluate the evolution of ovule gene expression 12 species of Solanum. Using the model described above, the authors find that gene expression similarity is correlated with the tree topology of protein-coding genes which is consistent with cis-acting effects of introgressed variation on expression. To my knowledge, this is one of the finest assessments of the evolution of gene expression in diverging species because instead of focusing only on reporting the results, there is an underlying model and a potential explanation of the reasons that lead to such patterns.
The manuscript is exceptionally well written, has an excellent level of scholarship, is well contextualized, has clear results, and the discussion points out the relevant caveats. Overall, the piece is a timely and excellent contribution to the literature and I am certain will have an important effect on how we understand the evolution of gene expression, speciation, and introgression altogether.

We thank the reviewer for their very encouraging comments.

I just have a few comments, all of which are rather minor and are meant to help the readability of the piece.

The colors in Figure 1 are hard to follow, especially for a color-blind person.

We now use a new color palette in Figure 1 which is hopefully more accessible.

Line 282. I think “thousands of quantitative traits’ refers to the expression of individual genes. I would rephrase this sentence to be more clear.

We have rephrased this sentence (now on lines 283-285):

“*We used previously generated introgression and gene expression datasets from the wild tomato clade, Solanum section Lycopersicon, to empirically evaluate the effects of introgression on thousands of expression traits.*”

Line 499. Elaborate on the rationale for this ordination.

We now provide additional clarification on the p-value calculation, in addition to correcting a written error in the formula (our analyses used the correct formula) (lines 516-521):

*“We assessed the rank i of the observed Q_3_ values among these resampled datasets, and a two-tailed P-value was estimated using the following formula:*

$$P=1-2*|0.5-(i/n)|$$

*where n is the number of observations (in this case, 10,000). This formula measures the deviation of the observed value from the center of the bootstrapped dataset, which would have a rank of 5000.”*
